# Cortical Intra‐Layer Hypersynchronization in Levodopa‐Induced Dyskinesia Mouse Model

**DOI:** 10.1002/brb3.70963

**Published:** 2025-10-20

**Authors:** Mohamed Khateb, Fadi Aeed, Shay Achvat, Shaked Ron, Yitzhak Schiller

**Affiliations:** ^1^ Department of Neurology Rambam Medical Center Haifa Israel; ^2^ Department of Neurology University Health Network (UHN), University of Toronto Toronto Ontario Canada; ^3^ Rappaport Faculty of Medicine Technion‐Israel Institute of Technology Haifa Israel

**Keywords:** levodopa‐induced dyskinesia | motor cortex | Parkinson's disease | synchronization

## Abstract

**Background:**

Levodopa (l‐dopa)‐induced dyskinesia (LID) is a common and difficult complication in Parkinson's disease (PD) patients. It may result from hyperactivation of the primary motor cortex (M1) due to hypoactivation of the basal ganglia (BG) output nuclei. Electrophysiological evidences are sparse, mainly due to technological limitations related to the poor ability to simultaneously acquire data from many neurons of the different involved regions. We exploited the Neuropixels technology to overcome these obstacles.

**Methods:**

Extracellular Neuropixels recordings were acquired from awake head‐restrained mice in wild‐type (WT), parkinsonian, and LID conditions. Activity was recorded from M1 and the motor striatum simultaneously and compared for each mouse in four conditions: control (WT with and without l‐dopa), hemiparkinsonian (6‐hydroxydopamine model), and LID states.

**Results:**

Neural firing rates in M1 were decreased in PD and increased in LID as expected. Focusing on the quiet periods, the firing rates between the different conditions were similar. LID was associated with cortical intra‐layer hypersynchronization, a phenomenon not previously described. The overall synchrony was significantly increased between neurons of Layers 2, 3, and 5 in M1 in LID compared to PD state. Inter‐layer cross‐correlation was increased in LID, compared to PD state, between Layer 5 of M1 and the striatum. These changes in functional connectivity were absent in WT mice receiving l‐dopa.

**Conclusions:**

Our single‐cell recordings from thousands of neurons provide insight into cortical network changes in LID. We found that LID is associated with intra‐layer hypersynchronization of neurons within the motor cortex, which may be an intrinsic network feature within the cortico–BG loop.

## Graphical abstract

Using Neuropixels technology, we obtained single‐cell data from thousands of neurons in the motor cortex and striatum of mice at different stages: WT, parkinsonian, and dyskinetic (LID). We found that LID is associated with intra‐layer hypersynchronization within the motor cortex, which may be an intrinsic network feature within the cortico–BG loop.

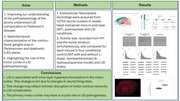

## 1 Introduction

Parkinson's disease (PD) is the second most common neurodegenerative disorder, with a lifetime incidence of 1%–2% (Kalia et al. 2015; Ascherio and Schwarzschild 2016). PD is usually treated by drugs that enhance dopaminergic neurotransmission, such as levodopa (l‐dopa) and dopamine receptor agonists. These anti‐parkinsonian drugs markedly improve motor performance, especially at the early stages of the disease. In contrast, in the more advanced stages of PD, patients only partially respond to the presently available pharmacological and neurosurgical treatments and develop severe adverse events to anti‐parkinsonian drugs (Kalia et al. 2015; Nelson and Kreitzer 2014; Verschuur et al. 2019). One important complication of l‐dopa and dopamine receptor agonists is the l‐dopa‐induced dyskinesia (LID). LID is common and difficult to treat (Fabbrini et al. 2007). They develop in almost all long‐standing PD patients treated with l‐dopa or dopamine receptor agonists and manifest as involuntary movements (Goldberg et al. 2002; Verschuur et al. 2019; Fabbrini et al. 2007; Bezard et al. 2001). Treatments like amantadine and deep brain stimulation demonstrated mild beneficial effects in reducing LID (Leta et al. 2019; Kwon et al. 2022). However, these approaches have limited efficacy and significant complications that limit their wide use. Therefore, novel antidyskinetic treatments are strongly needed, yet this aim cannot be accomplished without achieving a better understanding of the pathophysiology of LID.

At the cellular level, LID has been associated with a variety of mechanisms, including pulsatile stimulation of dopamine receptors and dysregulation of genes and proteins in downstream neurons, including in the nondopamine system, resulting in disruption in neural firing patterns between basal ganglia (BG) and cortex (Bezard et al. 2001; Obeso et al. 2000).

At the network level, hypoactivity of the BG output nuclei (globus pallidus internus [GPi] and the substantia nigra pars reticulata [SNr]) leading to hyperactivity of the motor cortex was suggested to be related to LID generation (Bezard et al. 2001). Electrophysiological evidences regarding LID are relatively sparse. It was proposed that LID is associated with a marked decrease in firing frequency of the GPi neurons both in parkinsonian monkeys (Bezard et al. 2001; Boraud et al. 2001) and in PD patients (Graybiel et al. 2000). Beyond changes in firing rates of neurons, changes in patterns of firing were also reported. Both D1 and D2 dopamine receptor agonists induce a shift for irregular firing patterns (Yang et al. 2021). Besides the “firing rate” and “firing patterns” hypothesis, the “ensemble model” was also proposed, relating dyskinesia symptoms to a distributed impairment involving many brain regions, mainly the M1 and the striatum (Yang et al. 2021; Girasole et al. 2018). In addition, other electrophysiological features including cortical gamma oscillations correlating with the abnormal movements were also reported (Güttler et al. 2021). More recently, genetic‐based neural perturbation techniques were used to model and decipher LID. For example, optogenetic modulation was applied to model LID in a cell‐type‐specific manner. This “optodyskinesia,” meaning dyskinesia induced by optogenetics, was achieved by in vivo optogenetic stimulation in hemiparkinsonian rats, inducing a D1‐dependent direct (Verschuur et al. 2019; Fabbrini et al. 2007) pathway activation in a similar manner of the dyskinetic state from chronic l‐Dopa (Hernández et al. 2017; Peng et al. 2022).

The primary motor cortex is located at a strategic position within the cortico–BG–cortical loop. The BG complex ultimately exerts its effects on motor function by modulating the activity of motor cortex, which in turn is responsible for generating motor output commands to the spinal cord and brain stem centers. Thus, ultimately LID is expected to impair the dynamics of the primary motor cortex even if the primary pathology is located at the BG. Indeed, accumulated evidence indicated that the motor cortex is involved in LID (Dupre et al. 2016; Halje et al. 2012). As previously elaborated, studies showed an increase in neural activity in M1 during LID in humans and in rodent models (Lindenbach et al. 2016; Cerasa et al. 2015; Ostock et al. 2011). However, other studies showed contradictory results in PD patients, indicating lower metabolic activity in M1 because of l‐dopa (Haslinger et al. 2001).

Previously, only a few studies attempted to explore the electrophysiological changes within M1 or the interplay between M1 and striatal structures during LID at the single‐cell level. In this study, we simultaneously recorded from M1 and the motor striatum in normal and parkinsonian mice without and with LID to investigate intra‐layer, inter‐layer, and inter‐regional synchronization changes related to LID generation.

## 2 Methods

### 2.1 Mice, Surgical Procedures, and Induction of Experimental Hemiparkinson

Experiments were performed on adult male (>8 weeks) wild‐type (WT) C57BL/6 mice. This study was performed in strict accordance with the guide for care and use of laboratory animals and approved by the Institutional Animal Care and Use Committee of Technion.

Every mouse underwent two surgical procedures. In the first, a headpost was created to enable head restraining during the handling and the acute recordings. Mice were anesthetized using isoflurane (4% for induction and 1.5%–2% during surgery). The mouse head was fixated in a stereotactic frame (Model 1900, Kopf Instruments, Tujunga, CA, USA). Prior to surgery, local anesthetic (lidocaine HCL 20 mg/mL) was injected at the scalp, and ketoprofen (5 mg/kg) and buprenorphine (0.1 mg/kg) were administered subcutaneously. Next, the skull above the right motor cortex was exposed, creating a small craniotomy. This craniotomy was covered with Kwik‐Cast for later probe insertions. After the first surgery of creating the headpost, mice were given a few days to recover, and during this time, handling was performed with four to five rounds of mice habituation for the recording setup in the awake head‐restrained manner. Next, the neuropixel probe was inserted, achieving the first extracellular awake head‐restrained recording from the WT state. During this recording, l‐dopa was systemically administrated, as will be discussed later. The craniotomy was covered again with Kwik‐Cast. The second surgery usually occurred a few days after the extracellular recording. Mice were anesthetized again as previously elaborated, and experimental hemiparkinsonism was induced by injecting the 6‐OHDA neurotoxin (3 mg/mL) into two locations within the motor striatum using a tilted glass pipette (Aeed et al. 2021; Assaf and Schiller 2019). For stereotactic 6‐OHDA injections, mice were anesthetized with isoflurane as previously mentioned. The 6‐OHDA‐containing solution was injected using a glass pipette held by a micromanipulator (MPC‐100, Sutter Instruments, Novato, CA, USA), and a microinjector (Narishige, Tokyo, Japan).

### 2.2 LID Induction

About 2 weeks after inducing the hemiparkinsonian state, l‐dopa was administrated (intraperitoneally) according to a protocol described in Cenci and Lundblad (2007). The l‐dopa was accompanied with a peripheral decarboxylase inhibitor to prompt the dyskinesia (Benserazide). l‐Dopa was administrated for 10 consecutive days, reaching a phenotypic plateau at the 10th day (Cenci and Lundblad 2007). In the following days, only maintenance doses were administrated. Evaluating the LID phenotype was performed by classic open‐field tests and by applying the Abnormal Involuntary scale (abnormal involuntary movement [AIM]). AIM was rated (by M.K. and F.A.) before l‐dopa injection and every 20 min after that until 1 h from the administration time. AIM score has five different possibilities: 0—no dyskinesia, 1—occasional signs of dyskinesia, which are present during less than half of the observation time, 2—frequent signs of dyskinesia that are present during more than half of the observation time, 3—dyskinesia is present during the entire observation time, but it is suppressible by external stimuli (e.g., sudden, loud opening of the lid of the cage), and 4—continuous dyskinesia that is not suppressible by external stimuli (Cenci and Lundblad 2007). It is important to state that there is no clear standardization regarding LID definition and evaluation in non‐primate experiments in the literature.

### 2.3 Extracellular Recording Using the Neuropixels Recording System

**FIGURE 1:**
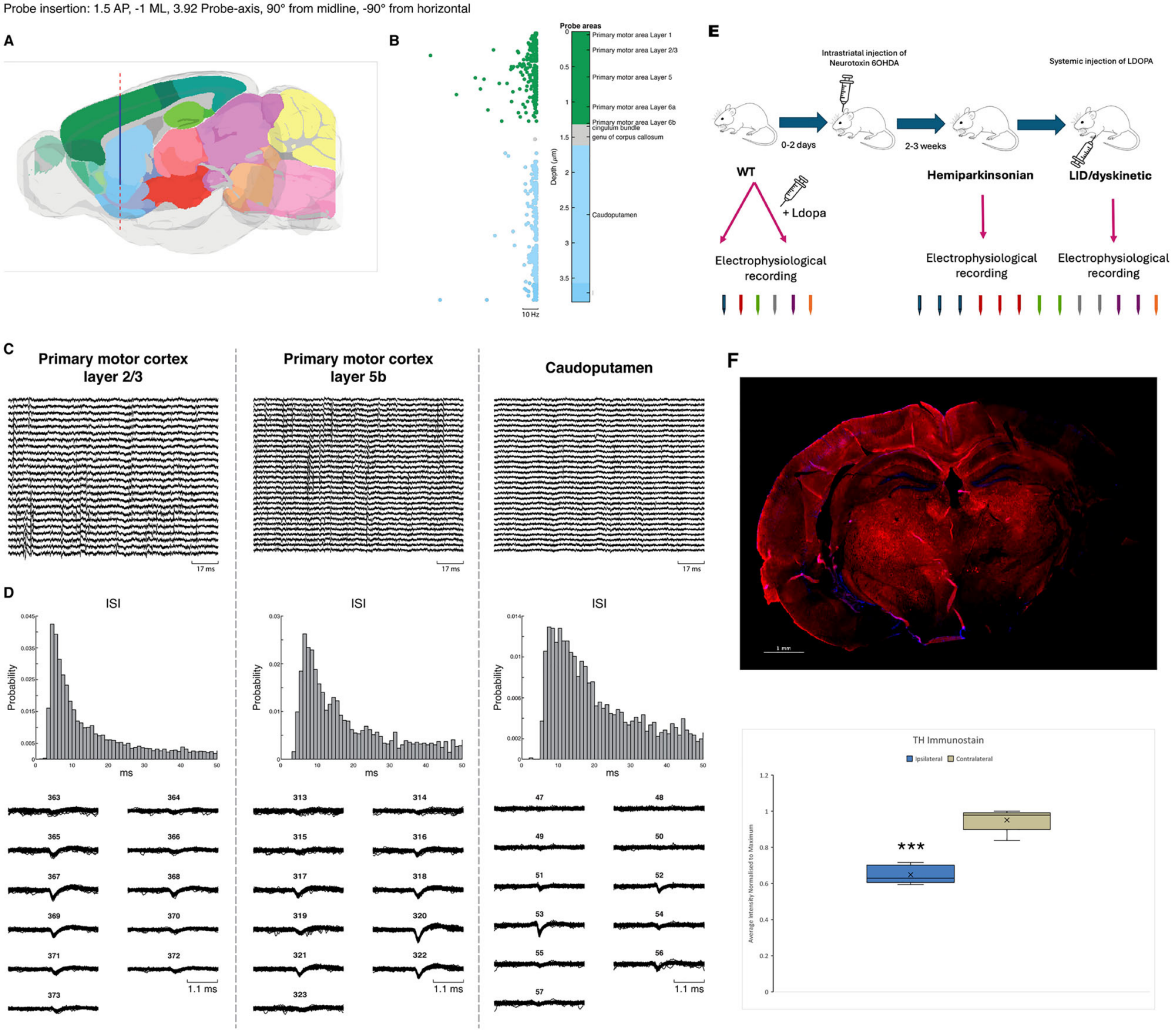
**Neuropixels recordings and experimental design**. (A) The trajectory of the neuropixel recording probe is demonstrated (superimposed on the brain atlas of Allen Institute). The probe was inserted at M1 and lowered to the depth of 3900 µm from the pial surface, achieving simultaneous extracellular recording from more than 600 neurons/clusters distributed along M1 and the striatum. (B) The probe's map is demonstrated with color‐coding for regions. Each dot represents a single neural cluster with information regarding firing rate frequency (green refers to cortical clusters, whereas blue is for striatal clusters; the scale bar represents a 10 Hz frequency). (C) A snapshot example of raw data activity at L2–3 of M1, L5 of M1, and the cpu. Three examples of clusters from the L2–3 and L5 of M1 and cpu. (D) The upper row represents the ISI (inter‐spike interval) graphs for each of the three examples. The waveform of each cluster is demonstrated as recorded from each of the different channels (each number refers to a different recording channel). (E) The experimental design is described; first mice had a surgical procedure for head post installation. Later, a wild‐type recording with the neuropixel probe was performed before and after l‐dopa administration. Few days later, the mice were intracranially injected with the neurotoxin 6OHDA, creating the hemiparkinsonic phenotype. About 2–3 weeks later the mice's overall condition began to improve, then LID was created by daily injection of l‐dopa intraperitoneally. After 10 days, the LID phenotype is completed. Multiple neuropixel recordings were performed at the PD and LID states in each mouse. The number of recordings performed for each mouse is color‐coded in each of the stages (WT and PD–LID) at the lower edge of this panel. (F) Histological data of a single PD‐LID mouse are presented. To quantify the TH stain differences between the injected and non‐injected side, we used ImageJ. After subtracting background fluorescence, 10 similar fields of view (FOVs) were assigned to the left and right sides of the striatum. Next, we compared the averaged intensity of each side, showing decreased staining in the ipsilateral side of the neurotoxin injection (*p* < 0.001, df = 5, two‐tailed *t*‐test).

The Neuropixels probe features 960 recording contacts arranged along a single 10 mm long and 70 µm wide shank (Hong and Lieber 2019; Jun et al. 2017). The probe can be programmed to actively switch from 384 independent recording channels. Thus, the Neuropixels probe can simultaneously record single‐unit activity from hundreds and sometimes more than a thousand neural clusters with very high temporal resolution and without depth limitations. In each recording session, the probe (Neuropixels 1.0 probe with caps) was inserted in the right M1 and lowered to a depth of 3800–3900 µm of the pial surface, achieving simultaneous recording from all the layers of M1 as well as the beneath striatum (Figure 1). Multiple recording sessions were performed from each animal (at WT before and after l‐dopa, PD, and LID states). First, a single acute recording was acquired during the WT stage, before inducing the hemiparkinsonian condition. An additional one to three separate acute recordings were performed after creating the PD–LID state, depending on the mice's survival, general state, and condition, and after providing the mice enough time to recover after each recording experiment (7–10 days). For data acquisition, we used built‐in software programs for the Neuropixels system. For preliminary data analysis such as filtering and spike sorting, we implemented external open‐source programs, based on MATLAB and Python, intended for Neuropixels recordings (e.g., https://github.com/cortex‐lab/KiloSort). The sorted spike trains were further analyzed using home‐written software in MATLAB (MathWorks, MA, USA). Clusters were accepted as single units if all the following criteria were met. The first criterion was if the cluster was labeled as “good” by the automatic KSLabel test of the open‐source Kilosort‐Python program. Additional criteria were according to our previous studies (Khateb et al. 2021). The second criterion was related to waveform shape consistency and stability throughout the recording. Units were excluded in case the average amplitude or half‐width of the unit changed significantly (ANOVA test) between the initial and last 20% of recorded spikes. The third criterion was inter‐spike interval (ISI) was >2 ms to avoid the noise and recordings from two different neurons. The fourth among the criteria was if the ISI distribution showed a smooth exponential‐like curve. The fifth and most important criterion is a statistical criterion of *p* < 0.05 (multivariate ANOVA) of cluster separation in the 2D planes and 3D spaces.

### 2.4 Data Analysis and Cross‐Correlation

Data were analyzed using open‐source programs for Neuropixels (MATLAB and Python). The automatic sorting of the neural clusters was manually verified or modified if needed for each of the obtained clusters. After the sorting, spike trains were extracted using home‐written software in MATLAB. To quantify synchrony, we calculated the pairwise cross‐correlation for all possible pairs in our recordings using MATLAB. Only eligible clusters were included in the analysis (previously elaborated). For each recording, we extracted a matrix containing the values of correlation between all possible pairs. Correlation between the signals of each of the clusters within a pair was calculated at different time lags, eventually selecting the maximal correlation value. For each option between L2–3/L2–3, L2–3/L5, L5/L5, L2–3/cpu, L5/cpu, and cpu/cpu, we calculated the averaged value of correlation in each of the states (WT, WT with l‐dopa, PD, and LID).

The correlation analysis elaborated above was applied only in quiet time periods of the recordings. These quiet windows were extracted from the video recordings as time intervals between each two consecutive movements of the mice with a confidence interval of 1 s after the offset and before the onset of movements. Times of movements were manually marked. The reason we chose to focus on these time intervals is to mitigate neurophysiological changes related to movements or changes in firing rates, ensuring that our results reflect intrinsic network changes in the cortico–BG loop. Considering the experimental setup of awake‐head‐restrained mice that were not trained for a specific task, a wide variety of non‐stereotypical movements were observed, making it difficult to separate and correlate neurophysiological changes according to specific movements.

Statistical analysis was performed using Microsoft Excel software and MATLAB, including the paired *t*‐test for comparison between different recording states (of the same animal) and multivariate ANOVA for cluster separation in spike sorting. Error bars in bar plots indicated the standard error of the mean (SEM).

### 2.5 Histological Preparations and Tyrosine Hydroxylase (TH)‐Specific Immunostaining

The histological samples were prepared as described in our previous work (Aeed et al. 2021). To quantify the TH stain differences between the injected and non‐injected side, we used ImageJ. After subtracting background fluorescence, 10 similar fields of view (FOVs) were assigned to the left and right sides of the striatum. Next, we compared the average intensity of each side (Figure 1F). The borderlines of each of the layers/regions were first defined according to the 3D model of probe insertion for neuropixels (Neuropixels trajectory explorer—based on the Allen CCF Mouse Atlas).

## 3 Results

### 3.1 The Behavioral Profile of LID Mice

**FIGURE 2:**
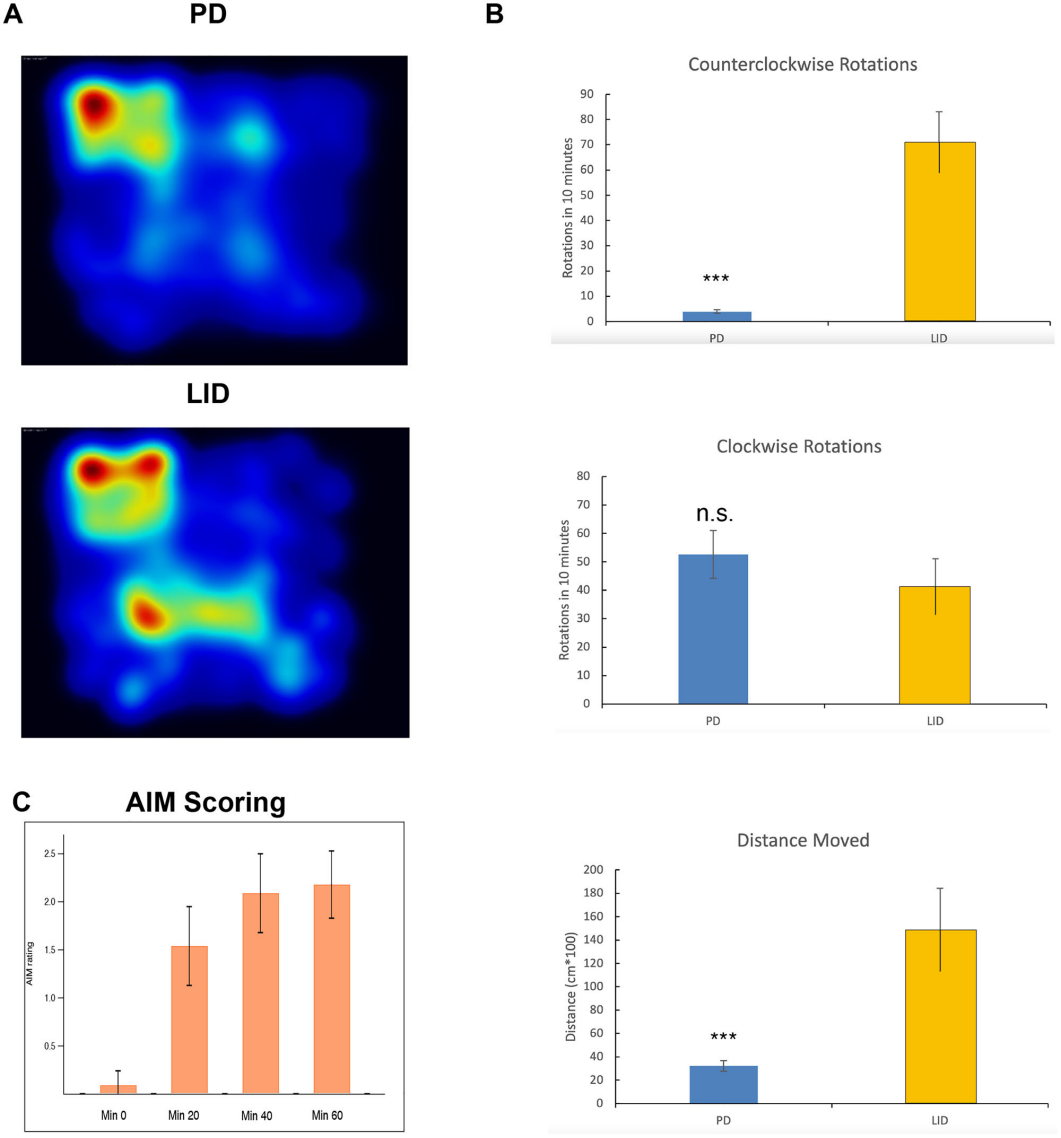
**Behavioral profile of PD–LID mice**. (A) A heat map of the open‐field test for a hemiparkinsonian mouse before and after inducing LID (upper panel—PD state, lower panel—LID state). (B) Bar graphs of averages of clockwise and counterclockwise turns and total distance moved (average ± SEM) of all the mice before and after inducing LID. Note the significant increase of the counterclockwise turns and in the distance in LID state compared with PD (*p* < 0.01 for counterclockwise and the distance analysis, *N* = 6 mice, df = 30). (C) The averaged AIM rating of all the PD–LID mice at times zero (just before l‐dopa administration), 20, 40, and 60 min (*p* < 0.01 between min 20 and min 0, df = 46, paired *t*‐test. AIM score had five different possibilities: 0—no dyskinesia, 1—occasional signs of dyskinesia, which are present during less than half of the observation time, 2—frequent signs of dyskinesia that are present during more than half of the observation time, 3—dyskinesia is present during the entire observation time, but it is suppressible by external stimuli (e.g., sudden, loud opening of the lid of the cage), and 4—continuous dyskinesia that is not suppressible by external stimuli. AIM, abnormal involuntary movement; LID, l‐dopa‐induced dyskinesia; PD, Parkinson's disease.

Compatible with previous reports, we found that parkinsonian mice are bradykinetic and tend to rotate ipsilaterally in relation to the lesioned side of the brain. Specifically, the average clockwise rotations in the PD state were 52.6 ± 8/10 min, close to what was shown in our previous paper (Assaf and Schiller 2019). Although the clockwise frequency was not significantly affected (possibly mildly decreased), the counterclockwise frequency dramatically increased due to the l‐dopa administration (Figure 2B). Counterclockwise rotations, per 10 min, were 4 ± 0.6 and 71.3 ± 11 in PD and in a fully developed LID state, respectively, *p* < 0.05, df = 30. Moreover, the overall distance measure increased dramatically in LID state, from 32 ± 5 in PD state to 148 ± 36 in LID state (*p* < 0.05, df = 30, Figure 2B). The AIM rating gradually increased in LID, as expected, reaching its plateau at 40–60 min since l‐dopa injection (Figure 2C).

### 3.2 LID Is Associated With Increased Firing Rates in the Primary Motor Cortex

From multiple recording sessions in each mouse, we obtained 10,753 reliable neural clusters across the cortico–BG loop. Of these 10,753 clusters, 1580, 4013, and 3711 were recorded from the L2–3 (0–350 µm from the pia), L5 (450–1000 µm from the pia) of M1 and the striatum (1500–3500 µm from the pia), respectively. The remaining 1449 clusters were recorded at borderline regions (between L2–3 and L5 or between the cortex and the striatum). These borderline‐recorded clusters were excluded from the analysis because of the difficulty in precisely determining their location.

**FIGURE 3:**
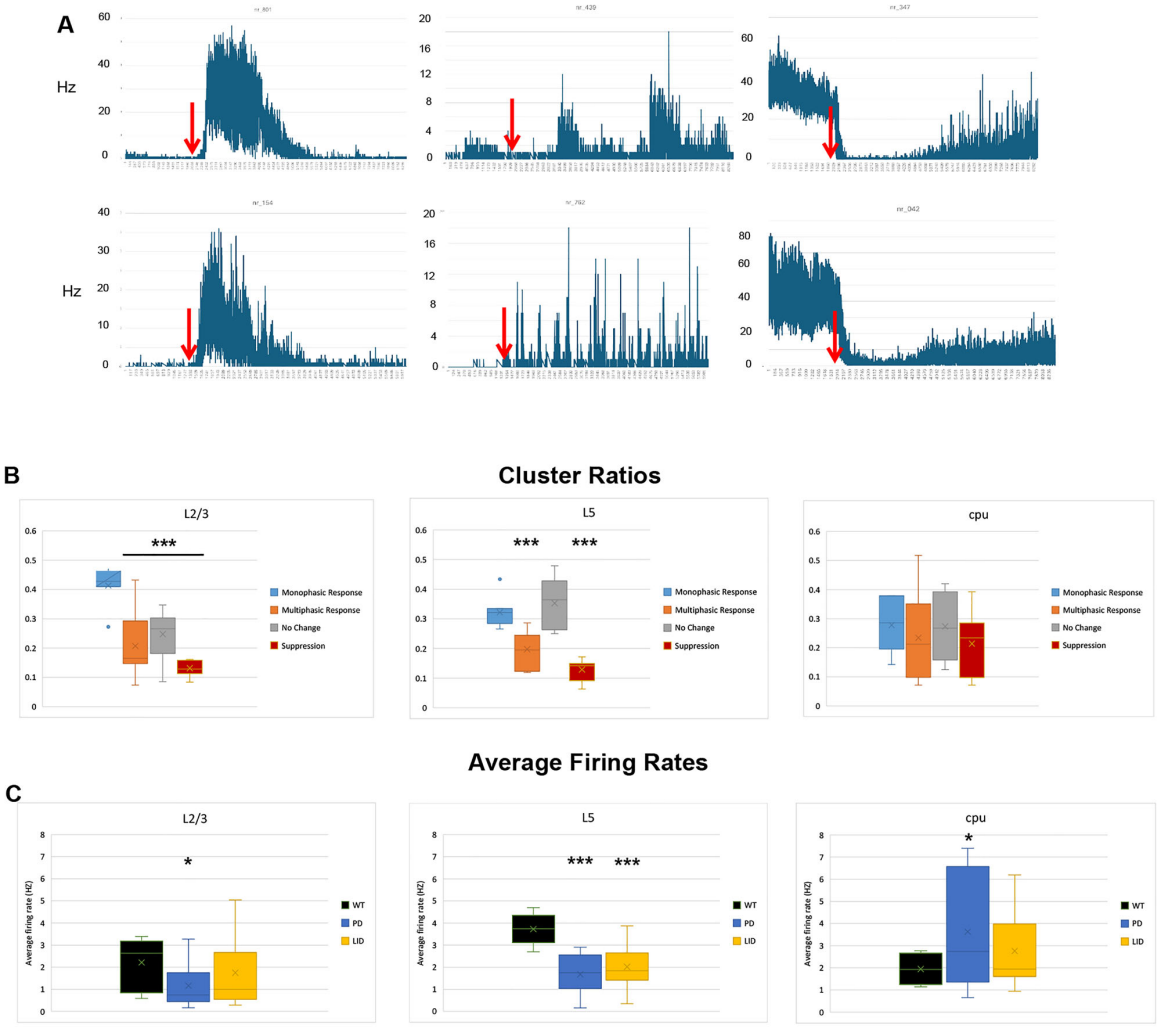
**Electrophysiological profile of PD–LID mice**. (A) Six rate histograms (time bin = 1 s) are presented for six different clusters at the cortico–BG loop. l‐Dopa administration is marked by the red arrows. The two clusters at the left side represent the monophasic responses; the middle two are bi/multiphasic responses, and the two clusters at the right side represent the suppression responses for l‐dopa. (B) Bar graphs (average ± SEMs) of the distribution of each of the clusters according to response pattern for l‐dopa in each of the regions: L2–3 of M1, L5 of M1, and the striatum (cpu). In L2–3, the proportion of monophasic responders was significantly higher than the other cluster types (*p* < 0.01, df = 24, two‐tailed *t*‐test). In L5, monophasic responder clusters and unaffected clusters were significantly more than the other cluster types (*p* < 0.001, df = 24, two‐tailed *t*‐test). In striatum, no significant difference was found between the cluster types. (C) Bar graphs (average ± SEMs) for averaged firing rates for each of the states (WT, PD, and LID) in each of the investigated regions (L2–3 and L5 of M1 and the striatum). We obtained in total 10,753 reliable neural clusters across the cortico–BG loop. Of these 10,753 clusters, 1580, 4013, and 3711 were recorded at L2–3, L5 of M1, and the striatum, respectively (two to four recording sessions from each of *n* = 6 mice). The firing rate in L2–3 of M1 increased by 50%, from 1.16 ± 0.31 Hz at the PD state to 1.75 ± 0.49 Hz at the LID state (1580 neural clusters, *n* = 6 mice, df = 24, *p* = 0.012, one‐tailed *t*‐test). In L5, firing rates were 1.68 ± 0.28 versus 2.02 ± 0.36 Hz at the PD and the LID states, respectively, *p* = 0.013 (4013 neural clusters, *n* = 6 mice, df = 24, one‐tailed *t*‐test). PD was significantly lower than the WT (*p* = 0.004, df = 17, one‐tailed *t*‐test). In striatum, PD averaged firing rate was 3.64 ± 0.92 Hz compared to 1.95 ± 0.23 Hz in WT (*n* = 6 mice, df = 17, *p* = 0.046, one‐tailed *t*‐test). The firing rate changed from 3.64 ± 0.92 to 2.76 ± 0.64 Hz at the PD and LID states, respectively (*p* = 0.05, *n* = 6 mice, df = 24, one‐tailed *t*‐test). LID, l‐dopa‐induced dyskinesia; PD, Parkinson's disease; WT, wild‐type. * is p < 0.05. ** is p<0.001.

Generally speaking, the averaged firing rate of neurons in M1 was decreased in PD compared to WT state (Figure 3C). In the LID state, the firing rate increased in M1. Specifically, the firing rate in L2–3 of M1 increased by 50%, from 1.16 ± 0.31 Hz at the PD state to 1.75 ± 0.49 Hz at the LID state (1580 neural clusters, *n* = 6 mice, df = 24, *p* = 0.012). A similar, yet less overt effect was observed at L5 of M1: 1.68 ± 0.28 versus 2.02 ± 0.36 Hz at the PD and the LID states, respectively, *p* = 0.013 (4013 neural clusters, *n* = 6 mice, df = 24). Nevertheless, this effect of LID‐induced increase in firing rates did not reach the previously recorded firing rate during the WT state in L5 of M1 (3.72 ± 0.249 Hz, *p* = 0.0016, df = 17). In L2–3 of M1, the averaged firing rate was similar between LID and WT states: 1.75 ± 0.49 Hz at the LID state versus 2.21 ± 0.41 Hz at the WT state, *p* = 0.53, df = 17 (Figure 3C).

In the striatum, an opposite trend was observed as the averaged firing rate increased in the PD state compared to the WT state. PD averaged a firing rate of 3.64 ± 0.92 Hz compared to 1.95 ± 0.23 Hz in WT (*n* = 6 mice, df = 17, *p* = 0.046). The firing rate in LID state decreased by 24% compared to PD. Specifically, the average firing rate changed from 3.64 ± 0.92 to 2.76 ± 0.64 Hz at the PD and LID states, respectively (*n* = 6 mice, df = 24, *p* = 0.05). No difference was found between LID and WT states (*p* = 0.23, 3711 neural clusters, *n* = 6 mice, df = 17) (Figure 3C).

LID state was correlated with changes in neural discharge rate at the cortico–BG loop. These changes in firing rate were classified into four main categories: monophasic positive response, bi/multiphasic positive response, no response, and suppression/negative response (Figure 3A,B). In Layers 2 and 3 of M1, 62% of the recorded clusters positively responded to l‐dopa, divided into 41.3% ± 2.2% responding monophasically and 20.7% ± 3.9% bi/multiphasically. The monophasically responding clusters increased their firing rate on average by 593% (from 0.32 ± 0.095 to 2.22 ± 0.293 Hz, *p* < 0.01, df = 24 [based on recording counts and not the unit's count]). An increase of 207% (from 0.243 ± 0.067 to 0.747 ± 0.138 Hz, *p* < 0.01, df = 24) was recorded for the bi/multiphasically responding clusters. The incidence of suppression response was 13.15% ± 1%. The average decrease in firing rate was by 80.7% (from 2.92 ± 1 to 0.563 ± 0.092 Hz, *p* < 0.05, df = 24). In Layer 5, 32.14% ± 1.9% and 20% ± 2% of clusters demonstrated mono‐ and multiphasic responses, respectively, whereas 12.9% ± 1.2% showed suppression. The average rise in discharge rate was by 351% and 486% for the mono‐ and bi/multiphasic responding, respectively, whereas the average suppression was by 74.5%. More specifically, a change from 1.028 ± 0.39 to 4.64 ± 1.42 Hz and from 0.363 ± 0.21 to 2.133 ± 0.57 Hz was recorded in the monophasic and multiphasic responding groups, respectively (*p* < 0.05 for both comparisons, df = 24). The suppression was from 6.61 ± 1.32 to 1.686 ± 0.29 Hz (*p* < 0.05, df = 24). In the motor striatum, of the 51% positively responding clusters, 28% ± 3.2% were monophasic and 23.4% ± 5.2% were multiphasic. In 21.4% ± 3.7% of clusters, suppression of firing rate was recorded. The LID‐induced average rise in discharge rate was 603% and 386% for the mono‐ and bi/multiphasic responding clusters, respectively, whereas the average suppression was 87.94%. Specifically, the firing rate changed from 0.377 ± 0.13 to 2.66 ± 0.506 Hz and from 0.875 ± 0.48 to 4.26 ± 1.09 Hz at the monophasic and multiphasic responding groups, respectively (*p* < 0.05 with df = 24 for both comparisons). Notably, the overall ratio of monophasic responding clusters was the highest in L2–3 compared with L5 and striatum (*p* < 0.01, df = 24 for both comparisons, Figure 3B). On the other hand, the striatum had the highest ratio of negatively responding clusters, *p* < 0.05 and df = 24 for both comparisons of striatum versus L5 and versus L2–3. No statistically significant changes were recorded between the three regions regarding the ratio of the bi/multiphasic responding clusters (*p* > 0.05, df = 24, Figure 3B).

### 3.3 LID Is Associated With M1 Intra‐Layer Hypersynchronization in Awake Head‐Restrained Mice

**FIGURE 4:**
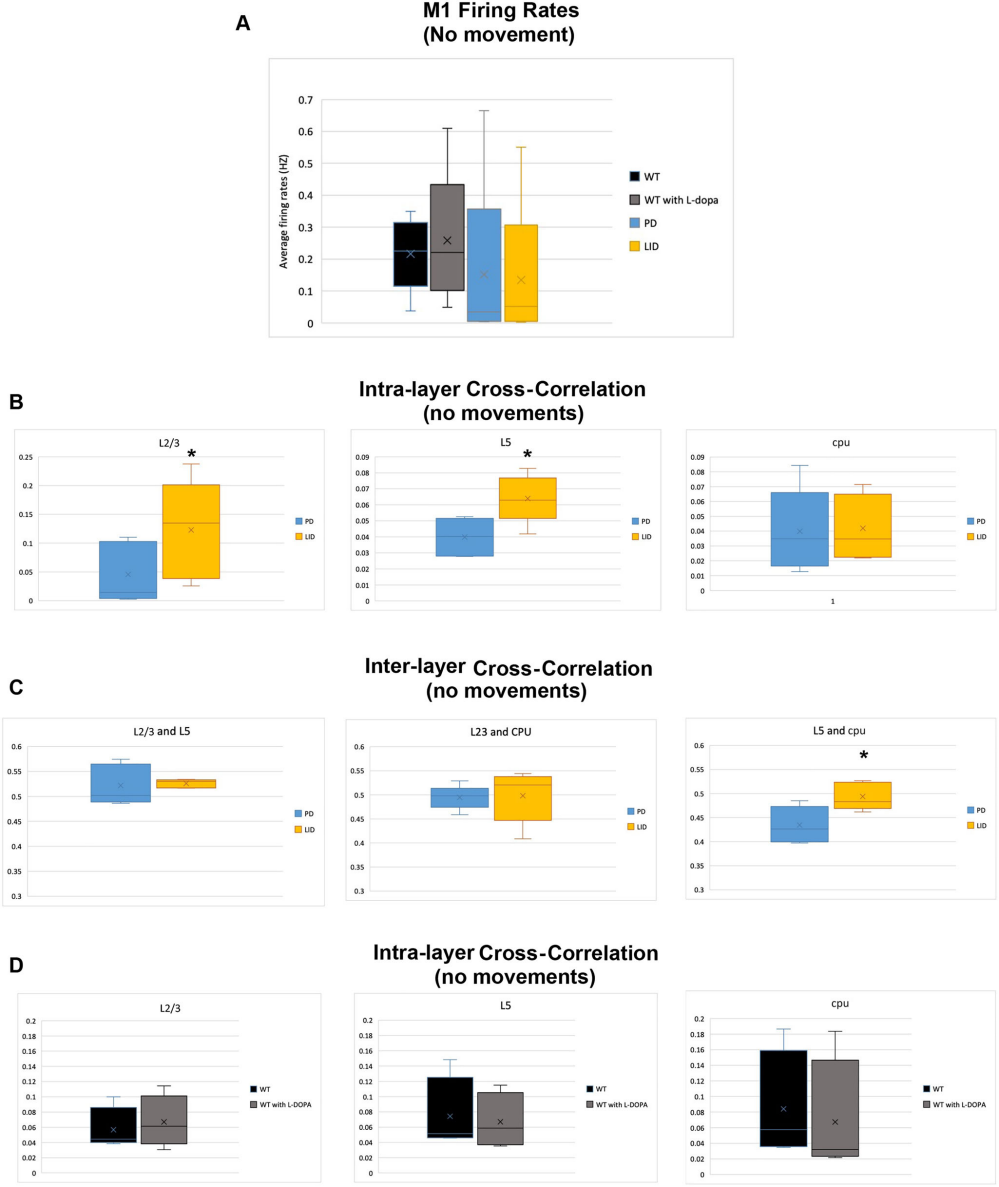
**Control experiments—electrophysiological profile of quiet times and for** l **‐dopa's effect in WT mice**. (A) Bar graphs (averages ± SEMs) of the firing rates during quiet times (recorded by the fast video camera) in WT (before and after l‐dopa administration), PD, and LID states (*p* > 0.05 for all comparisons, df = 10, 17, 24 for the different combinations of comparisons, *n* = 6 mice, one‐tailed *t*‐test). (B) Intra‐layer and intraregional averaged cross‐correlations (averages ± SEMs) restricted to quiet time periods for PD and LID states within L2–3 of M1, L5 of M1, and the striatum. Note the increase in correlation at LID state in L2–3 and L5 of M1 (*p* < 0.05, df = 24, *n* = 6 mice, one‐tailed *t*‐test). (C) Inter‐layer and inter‐regional overall cross‐correlations restricted to quiet time periods (averages ± SEMs) for PD and LID states between L2–3 of M1, L5 of M1, and the striatum (*n* = 6 mice). Only in L5 versus striatum was the averaged cross‐correlation significantly increased (*p* < 0.05, df = 24, *n* = 6 mice, one‐tailed *t*‐test). (D) Same as in (B) but in WT pre‐ and post‐l‐dopa injection. Note the lack of effect on cross‐correlation (*p* > 0.05, df = 24, *n* = 6 mice, one‐tailed *t*‐test). * is p < 0.05.

As previously elaborated in the methods part, we analyzed all the possible pairwise cross‐correlations throughout the quiet recording periods. Mice were monitored by a fast camera during the recordings to extract only the quiet time periods. The motivation behind focusing on the quiet time window in this analysis is to mitigate changes related to movement and/or firing rate of neurons. These time intervals were identified via a high‐frequency camera recording (∼200–300 Hz) as previously described in Section 2. As expected, in these time intervals, no significant change was recorded in the firing rate between the PD and the LID states (Figure 4A). The average firing rate in M1 was extremely low: 0.152 ± 0.117 and 0.135 ± 0.095 Hz during PD and LID states, respectively (*p* = 0.46, df = 24). Despite this lack of significant changes in firing rates between PD and LID states, the intra‐layer synchronization between neural pairs was increased (Figure 4B). In Layers 2 to 3 of M1, the averaged cross‐correlation was increased by 170% in LID compared to PD, from 0.043 ± 0.019 in PD to 0.113 ± 0.033 in LID (1580 neural clusters, *n* = 6 mice, df = 24, *p* = 0.03). Similarly, in Layer 5 of M1, a 60% rise was observed in the overall correlation, from 0.0399 ± 0.0048 during PD state to 0.064 ± 0.006 during LID state (4013 neural clusters, *n* = 6 mice, df = 24, *p* = 0.02). No significant changes were observed between PD and LID states in the dorsal striatum (3711 neural clusters, *n* = 6 mice, df = 24, *p* = 0.452). Inter‐layer/Inter‐region cross‐correlation showed a significant increase in the LID state between Layer 5 of M1 and cpu; from 0.434 ± 0.0155 in PD to 0.494 ± 0.0116 in LID, df = 24, *p* = 0.03. No significant differences were in the cross‐correlation between L23 and L5 of M1 or between L23 and cpu in PD and LID states (Figure 4C).

### 3.4 l‐Dopa Effect in WT Animals

To enhance the reliability of our results, we had two control conditions within the same mice: WT and WT plus l‐dopa. These conditions were investigated in the same mice before inducing the hemiparkinsonian and later the LID states, as previously described in the methods part. Applying l‐dopa to WT mice resulted in no change in the average firing rate in M1 during all the recording time: 3.34 ± 0.508 versus 2.61 ± 0.344 Hz before and after l‐dopa administration, respectively, *p* = 0.18, df = 10. Similarly, no significant difference in M1's firing rates was found during the quiet time periods (Figure 4A). In these time intervals, the averaged firing rate was low as expected: 0.214 ± 0.06 and 0.259 ± 0.1 before and after l‐dopa administration, respectively, *p* = 0.27, df = 10 (Figure 4A). There was no significant difference in the average cross‐correlation between pairs within or between M1's layers or between M1 and the striatum (Figure 4D). Specifically, the averaged cross‐correlation within L2–3 of M1 was 0.057 ± 0.013 and 0.067 ± 0.0155 in the pre‐ and the post‐l‐dopa time windows, respectively, *p* = 0.35, df = 10. Within Layer 5 of M1, 0.074 ± 0.022 in the pre‐l‐dopa window versus 0.067 ± 0.016 in the post‐l‐dopa window, *p* = 0.4, df = 10 (Figure 4D). Similar results were observed between each of the layers/regions in M1 and the striatum.

## 4 Discussion

Our results demonstrated cortical intra‐layer hypersynchronization in parkinsonian mice with LID. This effect is firing rate–independent and possibly reflects intrinsic changes in the cortico–BG loop. Moreover, this intra‐layer hypersynchronization effect was not observed in control WT mice receiving l‐dopa, suggesting the prerequisite of parkinsonism to achieve it. Our data support the view of LID‐related disruption of the intrinsic properties of cortical networks. Even though our results are based on the quite time periods and thus not directly associated with the abnormal LID movements, they reflect possible intrinsic disruption of cortical networks in the chronic LID state. These results are compatible with the previously reported intrinsic disruption of cortical networks in PD mice, challenging the classical view of PD/LID as extrapyramidal disorders (Aeed et al. 2021).

The motor cortex, and especially M1, is located at a strategic point within the BG–cortical loop thus making it a crucial region to possibly be involved in LID. A leading theory of the pathogenesis of M1 in LID is related to increased driving of the M1 network by the cortical–BG loop (Lindenbach et al. 2016; Cerasa et al. 2015; Ostock et al. 2011). Numerous previous studies addressed M1 changes in the parkinsonian state. These changes include neural firing rate (cell‐specific manner), increased beta activity of local field potentials, and electroencephalogram (Arbuthnott and Garcia‐Munoz 2016; Hooks et al. 2013; Brazhnik et al. 2012; Delaville et al. 2014). Parameters related to motor encoding were shown to be attenuated as well; pairwise synchronization of M1 neurons was found to be increased in MPTP‐treated monkeys (Goldberg et al. 2002; Pasquereau et al. 2016; McCairn and Turner 2015). On the other hand, data regarding electrophysiological changes in M1 in LID are less abundant.

Previously, intrinsic properties within the motor cortex were shown to be disrupted at PD state (Aeed et al. 2021). We show that this cortical disruption exists also at LID state, mainly as an intra‐layer hypersynchronization state, which is independent of firing rates. By taking advantage of the video monitoring and extracting the quiet time periods, our data demonstrated that this independency with activity level may suggest an intrinsic perturbation in M1 governing this effect in LID rather than disrupted inputs from the BG. Moreover, this effect is not the result of the l‐dopa itself, as we did not observe it when administrating l‐dopa to WT mice before creating the parkinsonian state. On the other hand, as our recordings were from the quiet time periods, we cannot confidently establish a direct behavioral–electrophysiological correlation between the abnormal dyskinesia movements and neural activity. Our results supported the idea that the chronic LID state is associated with intrinsic cortical changes and dys‐synchrony in its resting state despite the generation of the abnormal movements.

The effect of LID on neurons of M1 was shown to be cell‐type‐specific. Pyramidal neurons of the motor cortex consist of two types: Intratelencephalic (IT) and pyramidal tract (PT) neurons, based on their projections (Reiner et al. 2010; McColgan et al. 2020). IT neurons were found to show increased neural activity in LID, whereas the change in neural activity of the PT neuron is a matter of debate (Ueno et al. 2017, 2014). The effect on the GABAergic interneurons is even more ambiguous. This cellular‐selective effect of l‐dopa in M1 might be related to the results we observed of different patterns of changes in firing rates: monophasic, bursting/multiphasic, suppression, or non‐responsive.

At the network level, three main mechanisms have been suggested for LID. The first mechanism, named the “firing rate model,” proposes that decreasing the mean firing rates of the output nuclei of BG induces dyskinesia by causing overactivation of the motor cortex. Our data showed that firing rates were indeed increased in LID compared to PD state, yet without exceeding the previously recorded values during the WT state. This challenges the firing rate hypothesis, demonstrating its limitation to explain LID pathomechanism fully. The “firing pattern model” suggests that abnormal firing patterns may disturb the information processing in the BG, resulting in dyskinesia (Yang et al. 2021). Firing patterns and neural synchronization are strongly linked together (Kawaguchi 2001; Mann et al. 2005). Therefore, perturbations of the firing patterns can cause changes in cross‐correlations and synchronicity. An additional model, called the “ensemble model,” proposes that dyskinesia might reflect disturbances across many brain regions and that recruitment of activated neurons in the striatum correlates with the severity of the dyskinesia. Notably, a power increase in the high gamma frequency range was demonstrated in LID (Güttler et al. 2021; Belić et al. 2016). Our results, suggesting an intrinsic cortical intra‐layer dys‐syncrhony, favor the last model.

At the molecular level, LID was suggested to result from the imbalance between the direct and indirect pathways in the striatum. Both striatal dopamine D1 and D2 receptors are thought to be excessively stimulated, causing overshooting of activity in the direct pathway and an undershoot of activity in the indirect pathway. Whether the relationship between the observed intra‐layer hypersynchronization in the motor cortex and these changes at the receptor level is causative is still far from clear and needs more deciphering.

Our study has several limitations. First, we used the 6‐OHDA hemi‐PD model, which mimics a key feature of dopamine depletion pathology in the striatum, resulting in PD. This model differs from human PD disease in cardinal features such as the involvement of other nondopaminergic neurons in the neurodegeneration process. Nevertheless, the uptake of the neurotoxin is not selective by the dopaminergic neurons, as noradrenergic neurons and others might also uptake it and thus degenerate. This caused some of the previous studies to use desipramine, which is a noradrenergic uptake inhibitor that prevents noradrenergic neurons from uptaking the neurotoxin, thus producing a selective loss of dopaminergic neurons. In this study, as well as in previous papers of our group (Aeed et al. 2021; Assaf and Schiller 2019), we did not use desipramine because noradrenergic neurons were shown to degenerate in PD (Zarow et al. 2003), and some studies even suggested that this might happen before the degeneration of the dopaminergic neurons (Braak et al. 2004). Moreover, Cenci and Lundblad (2007) directly discussed this point, concluding that the protection of the noradrenergic cells is not necessary nor desirable when inducing the parkinsonian mice model. Moreover, human PD is usually bilateral and not unilateral. Third, the dopamine depletion pathomechanism in human disease is insidious rather than acute. Although the 6‐OHDA is a well‐established model for acute hemiparkinsonian induction, its reliability in chronic conditions such as the LID is obscure. Additional limitations are related to the head‐restrained setup rather than freely moving mice and the lack of cell‐type‐based technologies. As our focus was on the “quiet time periods,” the behavioral assessments of AIMs, or open‐field tests, were not correlated with the observed neuronal changes. The use of pairwise correlations as a reflection of functional connectivity is another limitation. Synchronized firing can result from common excitatory or even inhibitory inputs, including subcortical inputs such as thalamocortical afferents. Moreover, correlations can be context and state‐dependent. Lastly, the inability to identify specific cell types and correlate their responses with LID is an additional limitation of our studies. This crucial point might be addressed in the future using genetic‐based tools of neural identification.

To conclude, our study demonstrated, for the first time to our knowledge, that LID is associated with increased intracortical synchronicity, a phenomenon that is firing rate–independent. This conclusion is based on a huge amount of data recorded by Neuropixels from M1 and the striatum in awake head‐restrained hemiparkinsonic mice while monitoring with a fast camera. Viewing LID as a multisystem disease involving wide areas of the brain, including the striatum, the motor cortex, the pallidal–subthalamic network, thalamus, and the cerebellum, our data emphasize the importance of intrinsic changes within the motor cortex in the pathophysiology of the disorder.

## Author Contributions

**Mohamed Khateb**: conceptualization, investigation, writing – original draft, methodology, validation, visualization, writing – review and editing, formal analysis, project administration, data curation, supervision. **Fadi Aeed**: methodology, validation, visualization, writing – review and editing, software, formal analysis. **Shay Achvat**: methodology, software, formal analysis, data curation. **Shaked Ron**: methodology, software, formal analysis, data curation. **Yitzhak Schiller**: conceptualization, investigation, writing – original draft, writing – review and editing, project administration, supervision, resources.

## Ethics Statement

We confirm that we have read the Journal's position on issues involved in ethical publication and affirm that this report is consistent with those guidelines. Approval for this study was obtained by the Institutional Review Board at the Technion Institute.

## Conflicts of Interest

The authors declare no conflicts of interest.

## Peer Review

The peer review history for this article is available at https://publons.com/publon/10.1002/brb3.70963.

## Data Availability

Authors take full responsibility for the data, the analyses and interpretation, and the conduct of the research; they have full access to all the data; and they have the right to publish all data. Anonymized data not published within this article will be made available by request from any qualified investigator.
